# Functional Properties and Extraction Techniques of Chicken Egg White Proteins

**DOI:** 10.3390/foods11162434

**Published:** 2022-08-12

**Authors:** Zhe Li, Xi Huang, Qinyue Tang, Meihu Ma, Yongguo Jin, Long Sheng

**Affiliations:** National Research and Development Center for Egg Processing, College of Food Science and Technology, Huazhong Agricultural University, Wuhan 430070, China

**Keywords:** chicken egg white, protein, separation, purification, co-purification, poultry

## Abstract

Chicken egg whites contain hundreds of proteins, and are widely used in the food, biological and pharmaceutical industries. It is highly significant to study the separation and purification of egg white proteins. This review first describes the structures and functional properties of several major active proteins in egg whites, including ovalbumin, ovotransferrin, ovomucoid, lysozyme, ovomucin, ovomacroglobulin and avidin. Then, the common techniques (including precipitation, chromatography and membrane separation) and some novel approaches (including electrophoresis, membrane chromatography, aqueous two-phase system and molecular imprinting technology) for the separation and purification of egg white proteins broadly reported in the current research are introduced. In addition, several co-purification methods for simultaneous separation of multiple proteins from egg whites have been developed to improve raw material utilization and reduce costs. In this paper, the reported techniques in the last decade for the separation and purification of chicken egg white proteins are reviewed, discussed and prospected, aiming to provide a reference for further research on egg proteins in the future.

## 1. Introduction

Chicken egg white is popular in both the culinary and food industries as a convenient and readily available food ingredient. Egg white (EW) consists of about 88% water, 11% protein, 0.2% fat and 0.8% ash [1]. It is commonly found that EW is added in large quantities to pasta, baked goods, etc., mainly due to the good foaming and gelling properties contributed by the abundant proteins [2]. Moreover, egg white proteins (EWP) have antioxidative, antimicrobial, antiviral and/or immunomodulatory activities that are closely related to human health [3]. The prerequisite for employing the biological activity of EWP is the extraction of EWP from EW. Therefore, efficient extraction techniques are one of the keys to the research of EWP.

There are some difficulties in the direct separation of target proteins due to the complex composition of EWP, low concentration of target proteins and similar physicochemical properties of proteins [4]. Generally, EWP is subjected to a series of pretreatment processes such as filtration, homogenization, centrifugation and water dilution to obtain the raw material in the separation process. Then, suitable methods are selected to separate the target protein(s) from other substances. When there is only one target protein, the specific methods and parameters of protein isolation should be determined according to conditional tolerance of the target protein and its structures and physicochemical characteristics different from other proteins, such as solubility, molecular weight, polarity and charged situation.

The common methods reported in the literature mainly include precipitation, chromatography and membrane separation technology. These approaches are well established for use and allow for high separation efficiencies. However, there may be disadvantages of long operation time and/or high energy consumption. Moreover, the addition of chemical precipitants may cause reversible/irreversible denaturation of EWP and pose environmental hazards. In recent years, several novel, green and efficient extraction techniques have been developed to overcome the drawbacks of these commonly used processes and without causing environmental problems. In addition, a variety of methods are frequently used to jointly purify one or more proteins to improve egg utilization, reduce costs, and increase recovery and purity. The operational steps of co-purification are usually complex and time-consuming, and the methods used are relatively diverse. Moreover, the possible adverse effects of each step on all target proteins, such as yield loss and activity reduction, need to be considered. These difficulties need to be overcome in the development of co-purification.

In this review, the structures and functional activities of several major EWP are first presented as a prerequisite and are necessary for the separation and purification of EWP. Then, the separation and purification technologies of one or more target EWP are summarized, and the advantages and disadvantages of various methods are discussed, to provide ideas in the selection of the purification methods of EWP.

## 2. Structures and Functional Properties of Egg White Proteins

EWP are rich in functional activities. The prerequisite for applying the functional activity of proteins is to separate and purify them. Understanding the structural differences between various proteins is the reference for achieving protein separation and the basis for selecting the appropriate method. A brief description of the functional properties of the major proteins in EW is given below, and the basic characteristics (percentage of total protein, molecular weight (MW) and isoelectric point (pI)) are listed in Table 1 [5].

### 2.1. Ovalbumin (OVA)

OVA, the most abundant protein in EW, accounts for about 54% of the total EWP. OVA has a molecular weight of 45,000 Da and consists of a single chain of 385 amino acids with 105 titratable residues [6]. OVA is a mixture of three components (A1, A2 and A3 in the ratio 85:12:3) with slightly different electrical properties. Of these, A1 contains 2 mol of phosphoryl groups, A2 contains 1 mol of phosphoryl groups, and A3 contains no phosphoryl groups. All these phosphoryl groups are bound to the hydroxyl group of serine. The treatment of A1 and A2 with phosphatase resulted in phosphoryl group-free molecules [7].

OVA is precipitated by sulfate salting at the isoelectric point to obtain needle-like crystals of OVA. The high-purity refined proteins are obtained after recrystallization. These proteins are widely used to study the structure and properties of proteins and as experimental models for allergy [8]. As the most abundant protein in EW, OVA is a major contributor to the foaming, gelling and emulsifying properties of EW [9]. OVA not only has nutritional properties, containing all essential amino acids, but it also has excellent antioxidant activity. It has been reported in the literature that OVA has an inhibitory effect on the oxidation of phosphatidylcholine. The antioxidant activity was increased when OVA was covalently bound to polysaccharides [10]. Moreover, studies have shown that the food protein OVA might support immune defense for organisms by providing antimicrobial peptides [11]. Enzymatic digestion of OVA with trypsin and chymotrypsin revealed the antibacterial activity of these peptides, especially against *Bacillus subtilis* [12]. In addition, as one of the egg allergens, the separation and purification of OVA is an essential basis for exploring the mechanism and improving egg allergenicity [13].

### 2.2. Ovotransferrin (OVT)

OVT, also known as conalbumin, is an iron-binding glycoprotein that contains 0.8% mannose and is free of galactose and sialic acid. OVT accounts for about 12 to 13% of EWP and consists of 686 amino acids [14]. Each OVT molecule has two ligand centers located within the interdomain cleft of each globe. These two ligand centers can bind to metal ions such as Fe, Cu or Zn. The iron complex of OVT is stable to protein hydrolysis and thermal denaturation. It is red when OVT is combined with Fe^3+^ and has the maximum absorption value near 465 nm; it is yellow when OVT is combined with Cu^2+^ and has the maximum absorption value at 440 nm; the thermal denaturation temperature increases when OVT forms a complex with Zn^2+^, and the resistance to proteolytic enzymes and various denaturation treatments increases [15]. As members of the transferrin family, OVT and lactoferrin have similar properties and functions. OVT, however, is about 140 times more abundant in EW than lactoferrin in milk, making it an ideal candidate to make up for the shortage of lactoferrin [16].

OVT has a strong iron-binding capacity and therefore can be utilized as an iron supplement, antibacterial agent, antioxidant, and angiotensin-converting enzyme (ACE) inhibitor [17]. As an iron absorption enhancer, OVT, in two forms, the apo- (iron-free) and holo-form (containing iron at different saturation percentages), actively enhances iron absorption at the intestinal and gastric levels without accumulation and gastrointestinal irritation [18]. As an excellent candidate for iron supplementation, OVT can maintain the integrity of the gastrointestinal barrier to a greater extent and has a huge potential in dietary supplementation strategies [19]. The most widely accepted explanation for the antimicrobial effect of OVT is that OVT can bind iron required for the growth of iron-demanding microorganisms, thereby inhibiting growth, such as *Shigella dysenteriae*. Iron chelation-mediated iron deficiency by OVT also plays a key role in the suppression of *Salmonella*-induced egg infections. For other metal-demanding microorganisms, OVT has a similar inhibitory effect. Another explanation is that OVT causes membrane perturbation by direct contact with chelating divalent ions present on the outer membrane surface of gram-negative bacteria, resulting in bacterial inhibition. It has been reported that the inhibitory effect of OVT is closely related to the natural environment of EW (e.g., alkalinity, high viscosity, ionic composition and synergistic effects with other proteins) [20]. OVT exhibits antioxidant activity by scavenging superoxide radicals [21]. However, the antioxidant activity of natural OVT is relatively weak, and researchers have focused on the improvement of antioxidant activity to broaden its application [16]. On the one hand, antioxidant activity can be improved by adding natural plant extracts, such as vitamin C, caffeic acid and quercetin [22]. On the other hand, the products and derived peptides obtained from the hydrolysis of OVT exhibit stronger antioxidant activity than OVT. Therefore, the antioxidant activity of OVT can be better applied by selecting the appropriate enzyme-to-hydrolyze OVT to obtain antioxidant peptides. Similarly, the promod 278P enzyme hydrolysate of OVT showed high ACE inhibitory activity and has great potential for anti-cancer and anti-hypertensive drug applications [23].

### 2.3. Ovomucoid (OVM)

OVM is a glycoprotein containing sialic acid, about 4.5% mannose and about 1.5% galactose. OVM has nine disulfide bonds and does not contain free sulfhydryl groups. Disulfide bonds play an important role in the physiological activity of OVM. There are three regions in the structure of OVM, I, II and III, where region II inhibits trypsin. OVM binds to trypsin in a 1:1 ratio with a three-dimensional structure secured by three disulfide bonds in it [24]. The inhibitory activity disappears when the disulfide bond is broken, and the activity is restored upon re-oxidation. OVM has high thermal stability. The trypsin inhibitory activity of OVM is relatively stable when heated at pH 7 or below [25].

OVM can suppress bacteria by inhibiting the proteases needed for bacterial growth, thus protecting the developing embryo. In vitro, the trypsin inhibitory activity of OVM has significant applications. It can be added to prevent excessive hydrolysis after the addition of trypsin. In food, the use of egg-derived trypsin inhibitors has a higher safety profile than bovine-derived proteins that may pose a disease risk. In addition, OVM is commonly used as an alternative to serum trypsin inhibitors in cell culture. It can effectively inhibit trypsin after cell dissociation and prevent cell loss and death. The allergenicity of OVM is a significant area of research. OVM may play a more important role in the pathogenesis of egg white allergic reactions than other EWP [2]. Some modification experiments can effectively reduce the allergenicity of OVM, such as covalent bonding, superheated steam treatment and electrolysis [26,27,28]. Furthermore, OVM-derived peptides are biologically active. The selection of suitable enzymes for hydrolysis of OVM allows us to obtain active peptides for use as nutritional preparations or for food processing [29].

### 2.4. Lysozyme (LYS)

LYS is widespread in nature, the main sources include saliva, tears, mucus, human milk, and EW, among which LYS from EW is the most studied due to the easy availability of materials, low cost, and high content [30]. LYS from EWP has a small molecular weight (14.3 kDa), consists of 129 amino acid residues, and has an isoelectric point of 10.7. These characters clearly distinguish it from other EWP and are considered as important references for the selection of LYS separation methods [31]. Generally, isoelectric point precipitation and cation exchange work well for the separation of LYS from others. LYS also has high thermal stability and acid resistance. It is not denatured by heating at 100 °C for 1–2 min in acidic solution. The four-disulfide bond structure is partly responsible for maintaining the thermal stability of lysozyme [32]. In nature, LYS exists mainly in monomeric form. A reversible dimer is formed by changing pH, concentration and/or temperature causing a phase transition. The dimeric form of LYS exhibits therapeutic, antiviral and anti-inflammatory properties [33].

LYS is a typical alkaline nature protein, frequently used as a preservative and bactericide due to its ability to hydrolyze the peptidoglycan of the cell wall of bacteria [34]. However, gram-positive bacteria are better inhibited because they have more peptidoglycan components in the cell wall than gram-negative bacteria. Some modification approaches have been introduced to expand the antimicrobial spectrum of lysozyme, such as reductive modifications, polysaccharide coupling, enzyme modifications and oleoyl chloride modifications [35,36,37]. Heat denaturation leads to a gradual loss of LYS activity, but the antimicrobial effect on certain gram-negative bacteria is greatly enhanced [38]. LYS is popularly added in kimchi pickling, sushi, Chinese noodles, and cheese [39]. In the pharmacological field, LYS has an extensive use in wound-healing creams, eye drops and anti-cancer drugs [40]. Moreover, recent studies have shown that a non-peptide fragment (HL9) from human LYS has anti-HIV-1 activity in nanomolar concentrations [41]. In particular, as antiviral agents and immunomodulators, LYS, OVT and lactoferrin may be effective in the treatment or prevention of COVID-19 [42].

### 2.5. Ovomucin (OVN)

OVN is a glycoprotein consisting of an α-subunit (220 kDa, containing 10–15% carbohydrate) and a β-subunit (400 kDa, containing 50–65% carbohydrate), which are bound by disulfide bonds. OVN is more than 30% glycosylation and has a high molecular weight and high viscosity. These characteristics and its association with LYS and globulin give OVN excellent foaming ability and foam stability. Compared to bovine serum albumin, OVN has a much higher foaming ability. OVN is normally added to proteins as a foam stabilizer in the preparation of foods with the desired foaming capacity [43]. In addition, it was demonstrated that OVN has antibacterial, antiviral, antitumor and macrophage stimulating activities [44].

OVN is four times more abundant in the thick white than in the thin white. When the pH is around 9, the complexation of OVN and LYS is a cause of thinning of the thick white [45]. The extraction and study of OVN is valuable for the investigation of the mechanism of egg white thinning.

### 2.6. Ovomacroglobulin

Furthermore, ovomacroglobulin is a low level of globulin (0.5%) within EW that serves as a universally used protease inhibitor [46]. Its molecular weight is the second largest egg glycoprotein after OVN. Ovomacroglobulin contains four subunits of equal molecular weight (SDS-PAGE shows about 175 kDa), two of which are disulfide-bonded [47]. It has been shown that ovomacroglobulin has numerous biological activities, including antibacterial, anti-inflammatory, treatment of keratitis and inhibition of hemagglutinatio [48].

### 2.7. Avidin

Avidin is a strongly basic glycoprotein that is present in trace amounts in egg white (0.05%). Avidin is a tetrameric protein consisting of four identical subunits of identical amino acid composition and sequence (15.6 kDa and 128 amino acids each), each of which binds biotin with high affinity and specificity [49]. Avidin is irreversibly denatured at 70 °C, but the avidin–biotin complex is stable at 100 °C. The complex of avidin and biotin needs to be heated at 120 °C for 15 min to break down. The high affinity of avidin for biotin has been widely used in molecular biology, molecular recognition and labeling, enzyme-linked immunosorbent assays (ELISA), histochemistry and cytochemistry [50,51].

**Table 1:** Percentage of total protein, MW and pI of major egg proteins.

Protein	Percentage of Total Protein (%)	MW (kDa)	pI	Refs.
Ovalbumin	54	45	4.5	[6,7]
Ovotransferrin (conalbumin)	12–13	77	6.0	[14]
Ovomucoid	11	28	4.1	[24]
Lysozyme	3.4–3.5	14.3	10.7	[31]
Ovomucin	1.5–3.5	0.22–270 × 10^3^	4.5–5.0	[43]
Ovomacroglobulin (ovostatin)	0.5	7.6–9.0 × 10^2^	4.5–4.7	[46,47]
Avidin	0.05	68.3	10.0	[49]

## 3. Common Techniques

### 3.1. Precipitation

Precipitation is a method for separating proteins based on differences in solubility or isoelectric point. The precipitation methods of egg proteins mainly include salting-out, organic solvent precipitation, and isoelectric point precipitation. The precipitation of proteins in highly concentrated salt solutions to achieve separation is called salting-out, which is normally combined with a desalting process to obtain high purity proteins. Neutral salts are commonly utilized in the purification of EWP, which do not easily denature the proteins. The method of using organic solvents that are miscible with water to significantly reduce the solubility of proteins in water and precipitate them is named organic solvent precipitation. However, a high concentration of organic solvents tends to deactivate the protein and the volatility of organic solvents poses a food safety risk. The separation method based on the principle of minimum solubility of protein at isoelectric point is termed isoelectric point precipitation. Due to the similar isoelectric point of most EWP, isoelectric point precipitation has limited application and poor separation effect, which is frequently used in combination with other technologies. The precipitation methods are simple in operation and short in time consumption, but difficult to expand to an industrial scale.

Ammonium sulfate is commonly used to precipitate EWP because of high solubility, low temperature coefficient and low denaturation of proteins. OVT was precipitated with ammonium sulfate and critic acid and desalted by ultrafiltration. The yield of OVT was over 83% and the purity was over 85%. Since no organic solvent was used, OVT remained active and could be used for subsequent applications [52]. Polyethylene glycol (PEG) is also a commonly used precipitant. A method of separating OVA using PEG combined with isoelectric point precipitation with a purity of 95.1% was reported, achieving the processing of kilogram EWP solution in 2–3 h [53]. This method provided references for the industrial production of OVA. However, it is worth noting that OVA in solution is readily denatured and coagulated by exposure to new surfaces (e.g., shaking treatment), resulting in partial loss of activity. The precipitation method has been mature in the application of EWP separation and purification, but it is generally used in combination with membrane separation technology to remove salt or organic solvent. It has a poor effect when used alone, and the addition of precipitant may cause protein denaturation.

### 3.2. Chromatography

Chromatography is widely used in the separation of complex components. Its principle is to use the selective distribution of different substances in different phases to elute the mixture in the stationary phase with the mobile phase. Different substances in the mixture will move along the stationary phase at different speeds and finally achieve the separation effect [54]. Chromatography has the advantages of high separation efficiency, less use of raw materials, high sensitivity, fast analysis speed and simple operation. It is frequently used in the separation process of EWP and can be divided into ion exchange, gel filtration, affinity, and adsorption chromatography, according to the separation mechanism. These four methods are described and exemplified below.

#### 3.2.1. Ion Exchange Chromatography

Ion exchange chromatography is based on the difference in selectivity coefficients of the separated components using an ion exchanger as the stationary phase [55]. During EWP purification, a pre-loaded column was selected or a suitable ion exchange medium was prepared to achieve the separation based on the nature of the target protein. Among the major proteins of EW, LYS is commonly separated and purified by cation exchange chromatography because LYS is rich in basic amino acids and positively charged, whereas most other proteins are negatively charged.

A novel light-sensitive cation exchanger PNBCC was synthesized by a random copolymerization of chlorophyllin sodium copper salt, acrylic acid, n-butyl acrylate and N-isopropylacrylamide, which was used to purify LYS [56]. LYS activity increased by 16 times compared with original EWP solution. The magnetic chitosan (MCHT) beads were synthesized by phase inversion, and then grafted with poly (glycidyl methacrylate) (p(GMA)) via the surface-initiated atom transfer radical polymerization (SI-ATRP) [57]. In the presence of sodium sulfite, the epoxy groups of grafted polymers were modified into a strong cation exchange group, such as sulfonate groups. The magnetic cation exchange beads can be used for effectively purifying LYS. A cation exchange matrix with zwitterionic and multimodal properties was systhesized by a reaction sequence, coupling sulfanilic acid to a chitosan-based support, and the chromatography matrix was characterized physicochemically [58]. The chromatography matrix was recyclable, low in cost and easy to operate. Moreover, two diatom shells AQ1 and NP were used as cation exchange matrix, and the purification effect of LYS was evaluated, respectively [59]. The results showed that AQ1 diatom shell could be an excellent substitute for the cation exchange matrix in LYS purification. These reports demonstrated the realizability of ion exchange chromatography for the application of LYS separation, whereas ion exchange chromatography was rarely used alone for the purification of other EWP. It was more often used in combination with other methods, as exemplified in subsequent sections.

#### 3.2.2. Gel Filtration Chromatography

Gel filtration chromatography, also known as size exclusion chromatography or molecular sieve chromatography, is used to separate molecules of different sizes [60]. Water or a buffer solution is the mobile phase, and the molecular sieve effect of the network structure gel is utilized for isolating according to the molecular size of a separated object. No precipitants, such as organic solvents that can cause protein denaturation, are required in the using process. It is generally assumed that the separated proteins have the same symmetry, the protein with higher molecular weight is eluted first, and the protein with lower molecular weight is eluted later [61].

Gel filtration chromatography is suitable for separating proteins with large differences in molecular weight from other proteins, such as OVN and ovomacroglobulin in EW. During the separation process of OVN, a suitable pretreatment process should be selected to first reduce the viscosity of OVN and prevent the column from clogging. Wang, Tu, Tang and Shan [62] purified OVN by two-step salting-out and Sephacryl S-300 HR gel filtration chromatography with high antiviral activity maintained. The method overcame the difficulties of separating OVN caused by high molecular weight, high viscosity, easy degradation and other factors. Gel filtration chromatography also provide ideas for separating ovomacroglobulin. The ovomacroglobulin-rich precipitate was first obtained by PEG precipitation, then ovomacroglobulin was purified by two-step chromatography using the Q Sepharose Fast Flow anion-exchange column and the Sephacryl S-200 HR gel filtration column [48]. From further optimization on this basis, a one-step chromatographic method using a Sephacryl S-200 gel column could achieve 97.0 ± 0.3% purity of ovomacroglobulin [63]. This method simplified the complex chromatographic process and the whole purification process could be completed within one day. Gel filtration chromatography has the advantage of better separation, allowing for the entire separation in one column volume, and does not cause protein denaturation [64]. However, gel filtration columns are longer and have slower flow rates than other columns, resulting in longer elution time, higher column pressure, higher column material requirements and higher packing costs. Thus, gel filtration chromatography is generally used in conjunction with other methods to reduce separation time and prepare high-purity proteins.

#### 3.2.3. Affinity Chromatography

Affinity chromatography exploits the affinity between the substance to be separated and its specific ligand. Affinity chromatography allows for one-step purification instead of most time-consuming and complex methods [65]. Generally, bioligands are more specific, but they are more expensive and difficult to maintain activity in separation. In contrast, dye ligands are widely used, inexpensive and easily available, which can be immobilized on polymeric substrates by covalent bonding [66].

A novel functionalized graphene-based composite was prepared by covalent functionalization of continuously modified graphene oxide (GO) followed by chelation with nickel ions for the selective separation of LYS [67]. Reactive Red 120 was used as an affinity dye ligand to modify the surface of magnetic chitosan microspheres for improving their adsorption capacity to LYS [68]. These approaches could achieve recovery of about 90% but were more often used to concentrate and refine trace amounts of LYS. Currently, there were more reports on purification by affinity chromatography due to the significant affinity specificity of LYS, and fewer reports related to other EWP. Achieving repeatable stability of affinity-packed columns, thus reducing costs and improving purification efficiency, remains the focus of affinity chromatography in protein separation studies.

#### 3.2.4. Adsorption Chromatography

Adsorption chromatography employs a solid stationary phase, which takes advantage of the difference in adsorption properties of the solid adsorbent on the substance to achieve separation. Adsorption mainly refers to inter-molecular forces, such as hydrophobic forces, aromatic ring interactions, and even hydrogen bonding. Under certain conditions, the adsorbed material can leave the adsorbent surface, which is referred to as desorption. Adsorption chromatography is accomplished by successive adsorption and desorption.

A hydrophobic poly (hydroxyethyl-methacrylate-N-methacryloyl-L-phenylalanine) (PHEMAPA) bead was synthesized by suspension polymerization and then embedded into a poly (hydroxyethyl-methacrylate) (PHEMA)-based cryogel column. The PHEMAPA bead-embedded cryogel (BEC) column can be used to purify LYS by hydrophobic interactions, which is considered as an ideal column [69]. LYS was eluted in a small, packed bed in which 40 mM carbonate was used as a buffer (pH 12) and 0.5 M NaCl as an eluent, showing a better recovery of LYS from high viscosity EW [70]. Similarly, an affinity cryogel was prepared by covalent immobilization, the pore size and mechanical strength of the cryogel were increased, and high adsorption capacity for LYS purification was achieved [71]. STREAMLINE SP and SP-XL high density adsorbents were used as adsorbent carriers to optimize the adsorption conditions for LYS. These two adsorbents were well suited for the direct recovery of LYS from crude EW solution [72]. Adsorption chromatography has become an effective method for the purification of LYS due to its high purification efficiency, which is less reported in the purification of other proteins.

The effect of chromatography in the separation and purification of EWP is shown in Table 2. At present, chromatography is still the mainstream method for isolating high-purity proteins, although the operation is slightly more complicated and time-consuming.

### 3.3. Membrane Separation Technology

The applications of membrane separation technology in the separation of EWP have been reported in the literature. The most commonly used membrane separation technology is ultrafiltration. Ultrafiltration is to use a special membrane to selectively filter various solute molecules in the solution. When the liquid passes through the membrane under the action of certain pressure or centrifugal force, the solvent and small molecules penetrate and the large molecules are blocked. Dialysis is also a membrane separation technique, but it is more suitable for desalting or concentrating smaller volumes of sample solutions and has a longer operating time.

Due to the similar molecular weight of some proteins in complex components, the effect of direct membrane separation technology is poor. Therefore, membrane separation technology is frequently combined with other technologies, typically for sample concentration or for desalting and devolution after the addition of a precipitant. Amberlite FPC 3500 ion exchange resin and precipitation was firstly used to remove sequentially LYS, OVN, OVT and OVM to obtain OVA-rich precipitate. It was then precipitated with 35% saturation ammonia sulfate (final concentration), desalted and concentrated by ultrafiltration. Finally, ovoinhibitor was obtained by freeze-drying, with a purity of 90.8% and a yield of 70.0% [73]. Ovoinhibitor isolated in the study kept inhibitory activity on elastase, trypsin, alpha-chymotrypsin, and subtilisin. Membrane separation techniques are generally considered as an auxiliary method in the co-purification of multiple proteins, and examples are given in the Section Co-purification of Multiple Proteins.

## 4. Novel Methods

### 4.1. Electrophoresis

Electrophoresis is a method for separating target protein from other impurities based on the principle that charged molecules move towards electrodes opposite to their electric properties in an electric field. The main electrophoresis methods for EWP separation include isoelectric focusing and free-flow electrophoresis. LYS is normally bound to OVT to form a complex, Shimazaki, Ochi and Fujimura [74] separated and transferred LYS-binding proteins to membranes by non-denaturing two-dimensional electrophoresis (2DE). It was found that the LYS–OVT complex has lysis activity against both *Bacillus subtilis* and *Escherichia coli*. Furthermore, non-denatured gel isoelectric focusing was found to be capable of separating natural proteins that retained biological activity [75]. LYS was obtained at the top of the gel column at the end of the isoelectric focusing cathode. The results showed that the technology could effectively separate LYS in its natural state. The advantage of isoelectric focusing is high resolution, which can separate proteins whose isoelectric points differ by 0.01–0.02 pH units. Free-flow electrophoresis combined with gel filtration chromatography was successfully employed to isolate low-abundance LYS [76]. However, a special polyacrylamide-co-acrylic acid gel electrophoresis combined with mass spectrometry was used to identify the prepared LYS. Gel electrophoresis is normally used to identify and analyze proteins to determine the content and purity of proteins in samples. Polyacrylamide gel electrophoresis is the most commonly used. Because only small amounts of proteins are prepared, gel electrophoresis is used in combination with other separation techniques. In addition, free-flow electrophoresis and isoelectric focusing can be used in combination and is known as free-flow isoelectric focusing. This method provides the advantages of mild separation conditions, high recovery and high resolution. Wang et al. [77] constructed a homemade carrier ampholyte free-flow isoeletric focusing system by directed migration of H^+^ and OH^-^ provided by electrode solutions for separation of OVM, OVA, and OVT. This study was an effective attempt to solve the problem of the high cost of carrier amphoteric electrolytes. Electrophoresis can be used to separate EWP, but it is generally applied to extract small amounts of proteins.

### 4.2. Membrane Chromatography

Membrane chromatography is a technique combining membrane separation and chromatography. As a potential alternative to traditional packed-bed chromatography, membrane chromatography has the advantage of reduced hardware requirements, ease of operation and shorter processing time [78]. Polyacrylonitrile nanofiber membranes were prepared by the electrostatic spinning technique, which can be used for the purification of LYS. The one-step reaction was found to have a 90% capture efficiency and 47-fold purification factor [79]. Similarly, LYS was purified using tris (hydroxymethyl) aminomethane (P-Tris) functionalized polyacrylonitrile nanofiber membranes as affinity chromatography, achieving 93.28% recovery [80]. Traditional membrane adsorption applications are limited in a number of ways. The high-density functional group modification can improve membrane adsorption capacity. Accordingly, a novel high-capacity tetrazolium-functionalized weak cation exchange membrane was prepared [81]. The membrane showed better binding capacity for LYS and OVT compared to previously reported cation exchange membranes. Furthermore, Madadkar, Sadavarte and Ghosh [82] compared a laterally-fed membrane chromatography (LFMC) device with a strong cation exchange membrane with an equivalent conventional resin-filled column and found that the device had a significantly higher theoretical column number. The device could be used to separate a ternary model of a protein mixture consisting of OVA, OVT and LYS with significantly better resolution than conventional columns. Membrane chromatography exhibits advantages not found in individual membrane separation techniques and chromatography, and it has good prospects in the field of protein purification.

### 4.3. Aqueous Two-Phase Systems

The aqueous two-phase system is a novel method for the large-scale recovery of proteins. In recent years, the rapid development of the aqueous two-phase system has provided new possibilities for the industrial production of proteins and laid the foundation for downstream processing. PEG has excellent hydrophilicity and can cause protein molecules to aggregate and precipitate, and it also has significant applications in aqueous two-phase systems. The PEG/phosphate system could be used to purify avidin from EW, and in the most efficient small-scale aqueous two-phase system, the purification factor of avidin was 5.7 and the yield was 92% [83]. Pereira et al. [84] separated OVA using an aqueous two-phase system composed of PEGs of different molecular weights (PEG 400, 600 and 1000) and an aqueous potassium citrate/citric acid buffer (pH 5.0–8.0). The purification process could be achieved in one step. The method can achieve sustainable recovery of OVA at low cost and can be easily expanded to an industrial scale. Furthermore, ionic liquids were reported to purify EWP in aqueous two-phase systems. Ionic liquids refer to liquids composed entirely of ions, without the presence of electrically neutral molecules, stable liquid state, tasteless, odorless and pollution-free, convenient and environmentally friendly, and can be used as a good substitute for traditional organic solvents. Its application in the field of protein purification has grown rapidly in recent years. A novel acrylonitrile-butadiene-styrene copolymer consisting of a tetraalkylammonium-based ionic liquid and a potassium phosphate solution was prepared to recover 99% of the LYS from the ionic liquid-rich phase [85]. The proposed study not only broadened the scope of the application of ionic liquids, but also suggested novel options for solvents used in protein precipitation. The immaturity of the research on the aqueous two-phase system has caused it to be rarely used for commercial applications. In the future, the popular application of aqueous two-phase systems in the field of protein purification will require continuous efforts from researchers.

### 4.4. Molecular Imprinting Technology

Molecular imprinting is also known as molecular template technology. It utilizes template molecules to form an imprinted cavity within the polymer structure, creating shape recognizability and chemical selectivity for the target molecule, which has led to valuable applications in EWP separation [86]. A hollow embossed silica polymer with covalently bonded modified proteins on the surface and a novel membrane containing LYS recognition sites prepared by surface-initiated atom transfer radical polymerization both exhibited good selectivity for LYS [87,88]. Additionally, Wang, Dong and Bai [89] prepared a LYS molecularly imprinted polymer by embedding, which had twice the adsorption capacity of the non-molecularly imprinted polymer. Magnetic molecularly imprinted polymers are ideal for the specific separation of biomolecules. A novel core-shell nanocomposite was prepared for the specific recognition of LYS [90]. This composite had the excellent points of simple preparation, good selectivity, and high binding ability by easily accessing the template into the recognition chamber and being stable enough to be recycled many times without degradation in performance. Similarly, Xu et al. [91] prepared a magnetized molecularly imprinted polymer that could be used to specifically identify LYS. The results showed that the polymer could be repeatedly adsorbed four times without causing a significant decrease in adsorption capacity. Molecular imprinting technology has a promising future in purification because of its specific selectivity for target molecules. Currently, molecular imprinting technology is most frequently used in isolating LYS. For target proteins with a known structure and properties, molecular imprinting can be considered, which may be able to obtain a high purity. The application of novel methods in EWP purification is summarized in Table 3. Although the implementation of these methods was not yet mature and the reported effects of their application were less detailed, these studies indicated the possibility of novel methods in the separation and purification of EWP.

## 5. Co-Purification of Multiple Proteins

Currently, methods for the separation and purification of single proteins are well established, but the treatment of the remaining egg components after separation of the target proteins is a research problem worthy of consideration. There has been a tendency to establish a co-purification method, where several proteins in the raw EW are separated simultaneously by one or more methods to improve raw material utilization. Co-purification methods need to maximize the purity and yield and keep the activity of all target proteins. The purification steps are particularly complex and require several methods to achieve multiple purposes.

**Figure 1:**
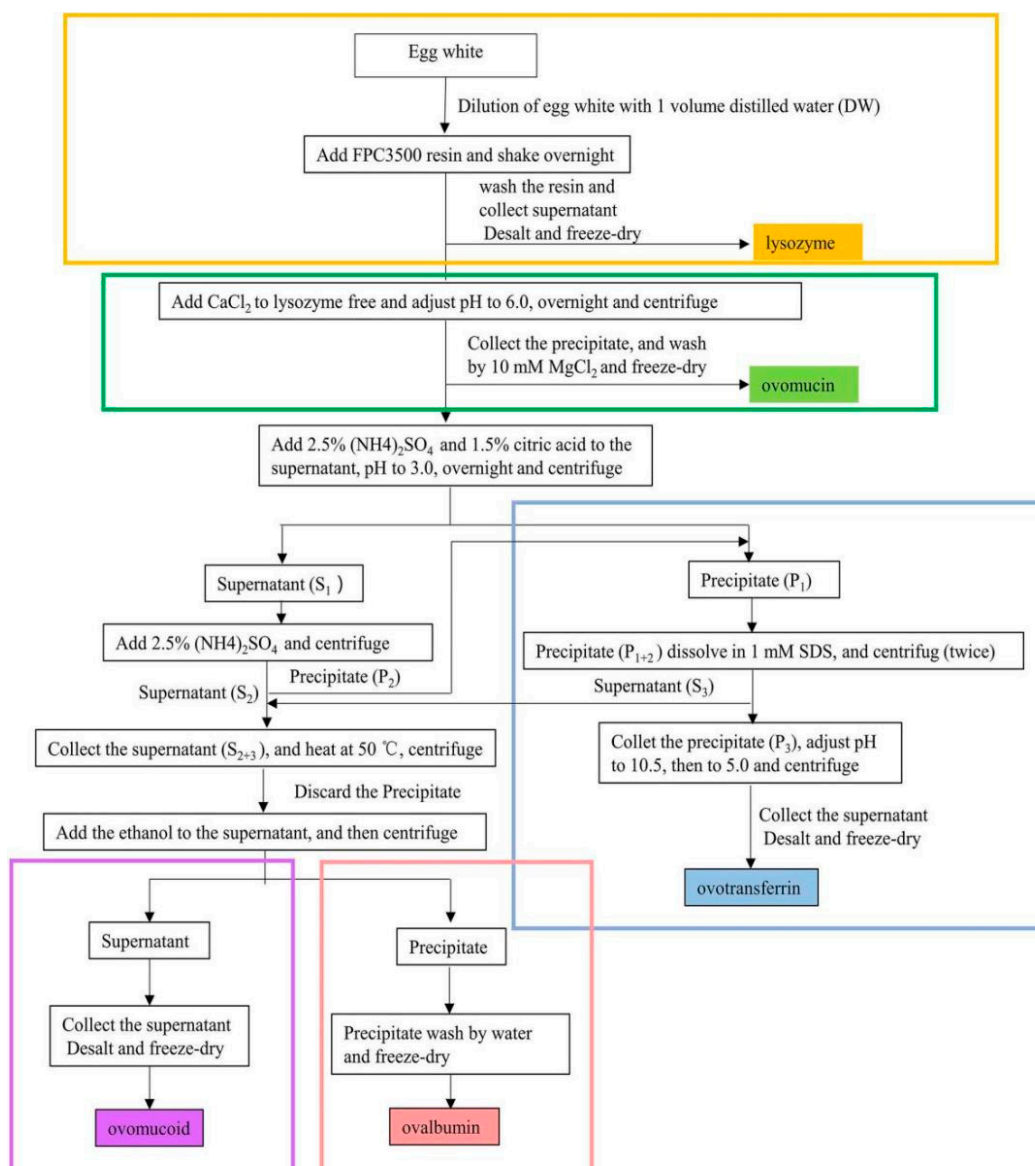
Flow chart of the co-purification process by Ji et al. [94].The different color boxes represent the key extraction processes for each protein.

The research team led by Ahn had an in-depth understanding of the subject. For instance, a method was proposed for the simultaneous purification of OVT and OVM [92]. The EW pretreatment solution was first precipitated with a combination of high concentration of ethanol and acidic salts. OVM was then further purified by heating at 65 °C for 20 min and centrifuged to remove impurities. The results showed that the yields of OVT and OVM were higher than 92% and 96%, and the purities were higher than 88% and 89%, respectively. ELISA confirmed that the activity of OVT was greater than 95%. In the same year, Abeyrathne, Lee and Ahn [93] sequentially utilized FPC3500 cation exchange resin, isoelectric point precipitation and salting-out assisted by centrifugation to separate LYS, OVA, OVN and OVT. The yields of both laboratory and scale-up preparations of OVN and OVA exceeded 98%, and the yields of OVT and LYS exceeded 82%. Similarly, the egg research team at Huazhong Agricultural University has conducted in-depth research on the protein co-purification. The resin was first washed successively with distilled water and glycine-sodium hydroxide buffer (0.1 mol/L, pH 9.3) to separate LYS from other insoluble and unadsorbed proteins. Then, OVT, OVA, OVM and OVN were separated by salting-out, isoelectric point precipitation and ethanol precipitation. As a major desalination method, ultrafiltration was necessary for further studies of proteins. The flow chart of the co-purification process is shown in Figure 1 [94]. The purities and yields of OVT, LYS, OVA and OVM were higher than 90% and 77%, respectively. The purity and yield of OVN were 72% and 75%, respectively. Geng et al. [95] used PEG graded precipitation combined with Q Sepharose Fast Flow anion exchange chromatography to separate LYS, OVT, OVA and ovoflavoprotein. The HPLC results showed purity of 91.84%, 94.55%, 96.45% and 88.16%, respectively. Additionally, Brand, Dachmann, Pichler, Lotz and Kulozik [96] separated LYS and OVT from high pressure homogenized EW using adsorption membranes as stationary phases for cation exchange chromatography. The purity of LYS was 96%, the yield was 99%, and the purification factor was 21, whereas the purity of OVT was 84%, the yield was 97%, and the purification factor was 5. Ma et al. [97] also used cation exchange chromatography to separate the four major proteins in EW. The yields of OVM, OVA, OVT and LYS obtained were 60.0%, 52.1%, 29.6%, and 90.2%, respectively, with high purity and maintaining antigenicity, and the method was suitable for the immunological characterization of these allergens. From these reports, it can be observed that most of the co-purification methods are now using ion exchange resins or chromatography, supplemented by precipitation methods. Moreover, LYS should be considered to be isolated first in the co-purification process of EW active substances, due to its positive charge and small molecular weight. All these combined methods have the advantages of easy operation, saving raw materials and a short purification cycle. In the future, co-purification will be the main research direction in separation of EWP. Researchers should build on current studies and develop novel methods to obtain as much active substances as possible.

## 6. Summary and Outlook

**Figure 2:**
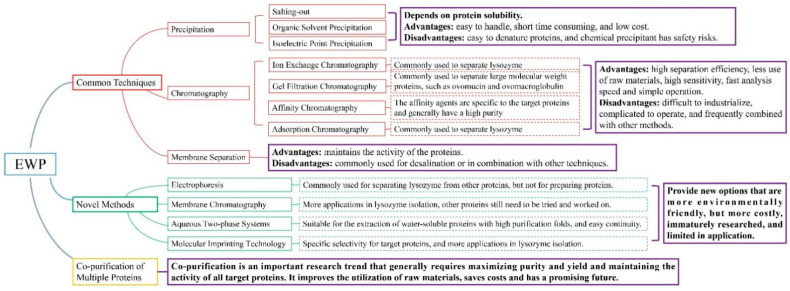
Summary of the separation methods of EWP.

The separation and purification methods of EWP should be determined according to the structures and characteristics of the target protein, such as molecular weight, solubility and isoelectric point. The methods should also meet the requirements of the laboratory scale or industrial scale, which requires higher purity on the laboratory scale, higher recovery rate, and lower cost on the industrial scale. Researchers should also consider the molecular activity of separated proteins. For example, the separation of LYS by organic solvent precipitation may lead to its denaturation and inactivation, which reduces its application value. Moreover, safety, cost and ease of operation should also be a concern. It is concluded that chromatography is the most widely used and mature technology in the separation and purification of EWP. However, chromatography is relatively cumbersome and takes a long time, and the cost of chromatographic column materials is high. Therefore, improving the recoverability of chromatographic columns, combining chromatography with other separation technologies, and adopting novel technologies to improve separation efficiency and shorten separation time are the key points of current EWP purification research. Furthermore, in the process of industrial production, researchers mostly use the combined separation method to separate a variety of egg proteins simultaneously, to improve the utilization rate of raw materials. As shown in Figure 2, the different methods apply to different EWP and have their own advantages and disadvantages. When selecting the separation and purification method of the target protein, researchers should fully understand its properties, and then integrate the characteristics of various separation technologies to determine the most suitable separation scheme. In the research of separation and purification of EWP, it is a vital trend to find a simpler, faster, more economical, greener, higher purity and recovery rate separation technology, and it is also an essential prerequisite to make full use of egg resources and widely apply them in food, biology, medicine and other industrial fields.

## Figures and Tables

**Table 2:** Applications of chromatography.

Method	Mechanism	Type	Target Protein	Yield	Purity	Activity	Refs.
Ion Exchange Chromatography	Differences in the ability of protein ions to compete with mobile phase for stationary phase surface charge positions	PNBCC	LYS	81.3%	-	Keep	[56]
		Magnetic chitosan (MCHT) beads	LYS	-	93%	Keep	[57]
		A cation exchange matrix with zwitterionic and multimodal properties	LYS	81.9%	86.5%	-	[58]
		AQ1 and NP	LYS	With AQ1 was 86%, and with NP was 82%	With AQ1 was 95%, and with NP was 90%	Keep	[59]
Gel Filtration Chromatography	Differences in molecular weight or molecular shape of proteins	Sephacryl S-300 HR gel column	OVN	3.02 g/kg fresh egg white	99.13%	Keep	[62]
		Q Sepharose Fast Flow anion-exchange column and Sephacryl S- 200 HR gel column	Ovomacroglobulin	37.76%	100%	-	[48]
		Sephacryl S-200 gel column	Ovomacroglobulin	62.5%	97.0 ± 0.3%	-	[63]
Affinity Chromatography	Differences in affinity between the substance to be separated and others with specific ligands	GO–PBA–IDA–Ni composite	LYS	90%	Electrophoresis pure	-	[67]
		Reactive Red 120	LYS	89.1%	80.7%	-	[68]
Adsorption Chromatography	Differences in the adsorption capacity of substances to be separated on the active adsorption center of the stationary phase surface	PHEMAPA BEC	LYS	-	Electrophoresis pure	-	[69]
		STREAMLINE Direct HST	LYS	94.3%	Purification factor of 15.7	Keep	[70]
		Low temperature copolymer gel	LYS	100%	-	-	[71]
		STREAMLINE SP and SP-XL	LYS	100% by SP vs. 93.78% by SP-XL	Purification factor of 26-fold by SP vs. 40-fold by SP-XL	Keep	[72]

**Table 3:** Applications of novel methods.

Method	Mechanism	Type	Target Protein	Yield	Purity	Activity	Refs.
	Differences in mobility of proteins in electric fields due to different charging of proteins when pH is at the isoelectric point or the non-isoelectric point	Non-denatured gel isoelectric focusing	LYS	-	-	Keep	[74]
		Free-flow electrophoresis	LYS	53.3%	80%	Keep	[76]
		Homemade carrier ampholyte free-flow isoeletric focusing system	OVM, OVA and OVT	-	-	-	[77]
Membrane chromatography	Using membranes as substrates to bind ligands, then separating proteins by adsorption, washing, elution, and regeneration	Polyacrylonitrile nanofiber membranes	LYS	87%	Purification factor of 47-fold	-	[79]
		Polyacrylonitrile nanofiber membranes functionalized with P-Tris	LYS	93.3%	Purification factor of 103.98-fold	Keep	[80]
		Novel high-capacity tetrazolium-functionalized weak cation exchange membranes	LYS and OVT	93%	-	-	[81]
		Laterally-fed membrane chromatography (LFMC) devices	The protein mixture consisting of OVA, OVT and LYS	-	-	-	[82]
Aqueous two-phase system	Differences in partition coefficients of substances between mutually immiscible two-aqueous phases. The partition coefficients depend on various interactions between the solute and the aqueous two-phase system, mainly electrostatic, hydrophobic and bio-affinity interactions	The PEG/phosphate system	Avidin	92%	Purification factor of 5.7	-	[83]
		The PEG/potassium citric acid buffer	OVA	65%	No other peaks in HPLC	Keep	[84]
		The tetraalkylammonium-based ionic liquid/potassium phosphate solution	LYS	99%	-	Keep	[85]
Molecular imprinting technology	Preparing specific molecularly imprinted polymers by simulating enzyme-substrate or antibody-antigen interactions for specific recognition of target protein	Hollow imprinted silica polymers	LYS	-	-	-	[87]
		Novel types of polymeric membranes	LYS	-	Separation factor of 23.08	-	[88]
		Molecularly imprinted polymers	LYS	98.2%	100%	-	[89]
		Novel core-shell nanocomposites	LYS	-	-	-	[90]
		Magnetized molecularly imprinted polymers	LYS	-	-	-	[91]

## Data Availability

Data is contained within the article.
